# Prenatal Detection of a Right Atrial Echogenic Mass: A Case Report of Hypertrophic Crista Terminalis

**DOI:** 10.3390/diagnostics16010017

**Published:** 2025-12-20

**Authors:** Mariangela Pati, Maria Paola Bonasoni, Andrea Musarò, Benedetta Petrachi, Francesco Di Dio, Elena Chesi, Francesco Leo, Giulia Barbato, Sonia Ricò, Giancarlo Gargano, Khush Shah, Lorenzo Aguzzoli

**Affiliations:** 1 Unit of Obstetrics and Gynecologic Oncology, Azienda USL-IRCCS di Reggio Emilia, 42122 Reggio Emilia, Italy; mariangela.pati@ausl.re.it (M.P.); andrea.musaro@ausl.re.it (A.M.); benedetta.petrachi@ausl.re.it (B.P.); lorenzo.aguzzoli@ausl.re.it (L.A.); 2 Pathology Unit, Azienda USL-IRCCS di Reggio Emilia, 42122 Reggio Emilia, Italy; 3 Neonatal Intensive Care Unit, Azienda USL-IRCCS di Reggio Emilia, Via Amendola 2, 42122 Reggio Emilia, Italy; francesco.didio@ausl.re.it (F.D.D.); elena.chesi@ausl.re.it (E.C.); francesco.leo@ausl.re.it (F.L.); giulia.barbato@ausl.re.it (G.B.); sonia.rico@ausl.re.it (S.R.); giancarlo.gargano@ausl.re.it (G.G.); 4 Department of Medicine, Lake Erie College of Osteopathic Medicine at Seton Hill, Greensburg, PA 15601, USA; ksshah0318@gmail.com

**Keywords:** hypetrophic crista terminalis (HCT), prenatal ultrasound (US), echocardiography

## Abstract

**Background and Clinical Significance**: The crista terminalis (CT) is a fibromuscular ridge located on the posterolateral wall of the right atrium, formed by the junction of the sinus venosus and the primitive right atrium. A hypertrophic or prominent CT (HCT) refers to a thickened or conspicuous configuration of this normal anatomical structure. In prenatal ultrasound (US) and/or echocardiographic assessments, HCT can mimic a right atrial mass, such as a tumor or a thrombus. **Case Presentation:** Herein, we describe a case of a fetal right atrial echogenic mass detected at 32 weeks, which remained stable through gestation and was confirmed postnatally as a likely HCT. No hemodynamic compromise, growth, or pathological sequelae were observed. **Conclusions**: Our case reinforces the importance of including atrial structural variants in the differential diagnosis of intracardiac masses, particularly when features favor stability and low risk. Serial imaging, avoidance of premature invasive measures, and careful counseling are key to appropriate management.

## 1. Introduction

The crista terminalis (CT) is a fibromuscular ridge located on the posterolateral wall of the right atrium, formed by the junction of the sinus venosus and the primitive right atrium [1,2].

Embryologically, the CT develops from the regression of the septum spurium and the fusion of the venous sinus, superior and inferior vena cava (SVC and IVC), and the original atrium during fetal development. This structure divides the right atrium into lateral and medial compartments and serves as the junction between the smooth-walled sinus venosus and the trabeculated right atrial appendage [1]. The CT is composed of working myocardial cells, pacemaker cells, and transitional cells, and functions as a critical zone for internodal conduction. Its high collagen content can contribute to slowed conduction, and age-related remodeling may be associated with abnormal conduction properties. The CT is recognized as a substrate for arrhythmias, including focal atrial tachycardia, ectopic beats, atrial fibrillation, and atrial flutter, making it a potential target for catheter ablation [1].

A hypertrophic or prominent CT (HCT) refers to a thickened or conspicuous configuration of this normal anatomical structure. In prenatal ultrasound (US) and/or echocardiographic assessments, HCT may be visualized and can mimic a right atrial mass, leading to diagnostic uncertainty and potential misclassification as a pathological lesion [1,2]. HCT can contribute to the appearance of a double-lobed right atrial appendage. In HCT, its broad-based insertion or exaggerated size may alter the contour of the right atrial appendage, potentially resulting in a double-lobed or bilobed appearance on imaging studies [3,4].

Recognition of HCT is crucial in the prenatal setting to avoid misdiagnosis of cardiac tumors or thrombi, which could result in unnecessary interventions or parental anxiety. Key imaging features that help differentiate HCT from pathological masses include its characteristic location on the posterolateral wall of the right atrium, similar echogenicity to adjacent myocardium, and lack of contrast enhancement [1,2,5,6]. Advanced imaging modalities such as transesophageal echocardiography (TEE) or cardiac magnetic resonance imaging (MRI) can further clarify the diagnosis if uncertainty remains after initial assessment [1,2,5].

The current medical literature indicates that HCT is a benign anatomical variant and not a pathological finding. There is no evidence to suggest that its presence in prenatal reports is associated with adverse outcomes or requires treatment. Its clinical significance is limited to its potential to be misinterpreted as a pathological mass, and awareness of its imaging characteristics is essential for accurate diagnosis [1,2,5]. The true incidence in prenatal reports remains undefined, as most data are derived from case reports and small series rather than large epidemiological studies [1].

Currently, only three cases of HCT have been reported in the prenatal setting [7,8,9].

Herein, we describe a case of a fetal right atrial echogenic mass detected at 32 weeks, which remained stable through gestation and was confirmed postnatally as HCT. No hemodynamic compromise, growth, or pathological sequelae were observed. This report adds to the literature the importance of considering benign atrial variants in the differential diagnosis of fetal intracardiac masses. Greater awareness and documentation of such cases may reduce misdiagnosis, and unnecessary interventions.

## 2. Case Presentation

A 29-year-old gravida 1, para 0 patient was first referred to our department at 28 weeks’ gestation due to suspected IUGR. The pregnancy up to that point had been unremarkable, including a normal first-trimester screening (ETS) and a normal non-invasive prenatal test (NIPT).

At 28 weeks’ gestation, the patient was referred to our prenatal diagnosis center due to restricted fetal growth, with biometric measurements at the 10th percentile.

At 32 weeks, a third-trimester US revealed a hyperechoic, well-defined, round mass in the fetal right atrium measuring 3.7 × 2.9 mm. The mass extended from the SVC to the IVC, along the posterior and lateral atrial wall. Cardiac blood flow remained normal, and no associated structural cardiac anomalies were identified.

**Figure 1:**
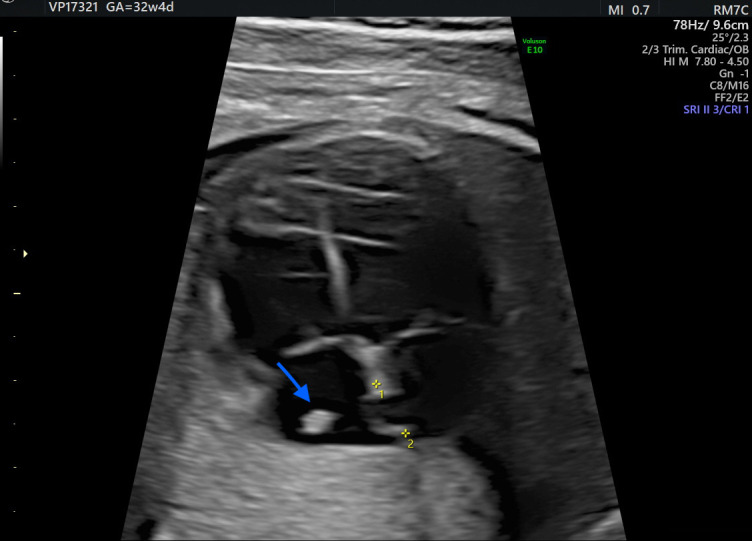
4-Chamber view at 32 + 4 week scan, demonstrating untypical hyperechogenic mass (3.7 mm × 2.9 mm) (arrow) being diagnosed in the later course as a prominent crista terminalis. 1, 2: interatrial septum.

**Figure 2:**
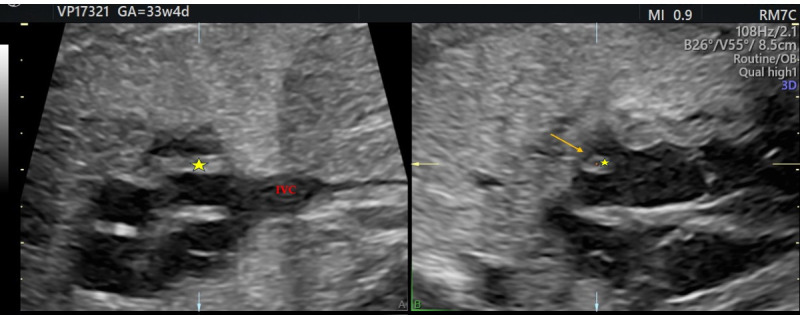
(**A**,**B**) Hypertrophic crista terminalis, ultrasound at 33 weeks + 4 days: a hyperechoic, round mass was seen in the right atrium (yellow arrow), which on 3D reconstruction appeared as a membranous structure traversing the atrial cavity (star). IVC: inferior vena cava.

Additionally, a thin membranous structure was observed traversing the right atrium and inserting at the superior aspect of the interatrial septum. This structure did not impede intra-atrial flow. Doppler evaluation of the ductus venosus demonstrated normal waveforms The differential diagnosis included HCT, atrial myxoma, rhabdomyoma (less likely), and intra-atrial thrombus. However, serial US executed during the remainder of the pregnancy displayed no changes in the size or characteristics of the mass (Figure 1 and Figure 2). Therefore, HCT was the favored diagnosis and no prenatal or postnatal MRI was planned. The US scans in pregnancy were defined in accordance with the most up-to-date evidence as there are no documented prenatal consequences of HCT [1]. Although there are no established guidelines for HCT, the American Heart Association and the American Society of Echocardiography (AHA/ASE) recommend repeated imaging in 2–4 weeks of the initial examination is incomplete or a progressive lesion is suspected, and the management must be individualized to the specific case [10].

The patient delivered at term as there was no risk of preterm induction. A female neonate weighing 3428 g was born with Apgar scores of 9, 10, and 10 at 1, 5, and 10 min, respectively.

**Figure 3:**
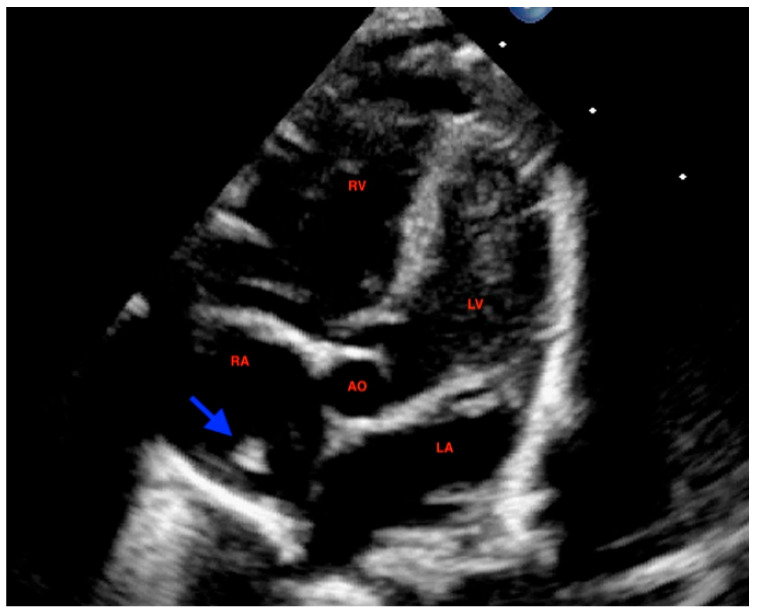
Postanatal apical 5-chamber view: echocardiography at 72 h of age displayed a persistent hyperechoic mass (arrow) within the right atrium compatible with hypertrophic crista terminalis. The structure showed no variations in growth compared to previous scans. RA: right atrium; RV: right ventricle; LA: left atrium; LV: left ventricle; AO: ascending aorta.

At 72 h of life, postnatal transthoracic echocardiography demonstrated a persistent hyperechoic mass in the right atrium, not grown and not modified compared to previous scans, and interpreted as HCT. There was no evidence of right atrial inflow obstruction, and no mass growth was observed (Figure 3).

The follow-up imaging was scheduled every 3 months and stopped at 7 months of age. At that time, the infant was still in good health with no significant clinical manifestations.

The follow-up imaging was defined in accordance with the most up-to-date evidence, as there are no documented prenatal consequences of HCT [1]. Moreover, there are no established guidelines recommending follow-up in the US specifically for HCT. The American Heart Association and the American Society of Echocardiography (AHA/ASE) do not recognize it as a pathological prenatal finding. Routine follow-up is unnecessary for normal variants, but if the initial study is incomplete or a progressive lesion is suspected, repeat imaging in 2–4 weeks may be appropriate according to guidance [10].

## 3. Discussion

We presented a case of a fetal right atrial echogenic mass discovered late in gestation, which remained unchanged and was confirmed postnatally as a benign anatomical variant: HCT. The pregnancy was overall regular and the fetus showed no other relevant abnormalities. After birth, the newborn was in good clinical condition and scan follow-up stopped at 7 months of age.

HCT typically appears as a reflective echogenic structure within the right atrium on imaging. Careful tracing of the CT to its anatomical origin, along with the use of TEE, can help distinguish it from other cardiac abnormalities. Case reports have described incidental findings of HCT in adults undergoing echocardiography for evaluation of arrhythmias, dyspnea, or congestive heart failure [2,11].

As a complementary tool to echocardiography, cardiac MRI is highly effective in diagnosing HCT, as it provides multiplanar imaging and superior tissue characterization. HCT is seen as a muscular ridge on the posterolateral wall of the right atrium, with signal intensity and enhancement patterns similar to adjacent myocardium and no contrast uptake, which are key features distinguishing it from neoplastic or thrombotic lesions [1,12].

In the prenatal setting, initial diagnosis is usually in the late second or early third trimester; HCT presents consistently as a hyperechogenic mass without progression throughout gestation or any color Doppler perfusion. The axial four-chamber view is the most relevant US plane for diagnosing HCT in the prenatal setting. This view allows direct visualization of the right atrium, including its posterolateral wall, where the crista terminalis is anatomically located. The American Institute of Ultrasound in Medicine, as part of its practice parameters, specifically recommends the four-chamber view for fetal cardiac assessment in the first trimester, as it enables evaluation of chamber symmetry and identification of intracardiac masses or unusual structures such as a prominent crista terminalis [13].

Additionally, the three-vessel and trachea view (3VT), which is a transverse plane through the upper mediastinum, can help delineate the relationship of the superior vena cava and right atrial appendage—key landmarks for localizing the crista terminalis [14]. The crista terminalis originates near the superior vena cava and divides the right atrial appendage from the sinus, making these views essential for accurate anatomical assessment.

Although the AHA emphasizes that fetal echocardiography is the gold standard, MRI may serve as a complementary modality when US assessment is suboptimal, such as in cases of maternal obesity, unfavorable fetal position, or oligohydramnios [10].

To the best of our knowledge, only 3 prenatal cases have been described in the current literature.

Pérez-Muñuzuri et al. [7] described a third-trimester fetus with HCT detected by US and confirmed after birth. The lesion remained stable with preserved blood flow, and additional findings included a right-atrial septal aneurysm and a Chiari network.

Evong et al. [8] reported HCT first identified at 20 weeks’ gestation and still visible at 29 and 33 weeks, occupying the right-atrial posterolateral wall up to the venous junction without affecting blood flow. Postnatal echocardiography confirmed HCT, and the child remained asymptomatic.

Bhatia et al. [9] documented a 3.4 × 4.6 mm HCT at 23 weeks, stable throughout pregnancy and similar in echogenicity to surrounding myocardium. Pediatric follow-up showed regression of the lesion.

Generally speaking, prenatal detection of intracardiac masses must prompt detailed evaluation, parental counseling, and decisions about surveillance or intervention.

Differential diagnosis must take into account true cardiac tumors (such as atrial myxoma, rhabdomyomas, fibromas, teratomas, hemangiomas), intracardiac thrombi, and benign anatomical variants or pseudomasses (HCT, Chiari network, muscular trabeculations) [15,16]. Misinterpreting a benign variant as pathological carries risks of unnecessary invasive testing, anxiety, or even pregnancy termination.

In the present case, a tumor was excluded as the lesion was solitary, stable and not growing during gestation, there was no arrhythmia, no hemodynamic impact, and the cardiac anatomy was otherwise normal. Postnatal confirmation of stability and lack of sequelae further argue against tumor origin.

Intracardiac thrombi in the fetal heart are exceedingly uncommon unless predisposing conditions exist (arrhythmias, intracardiac instrumentation, maternal–fetal clotting disturbances). Thrombi may appear as echogenic, avascular intracavitary masses that may resolve or evolve. In our case, the lack of risk factors and the stable imaging over many weeks make a thrombus unlikely.

Fetal intracardiac thrombi appear on US as discrete, well-defined echogenic masses within a cardiac chamber, usually larger and more irregular than echogenic intracardiac foci and not moving with the valve apparatus. They may be mobile or attached to the endocardium, create filling defects, appear in multiple planes, and show no flow or twinkling on Doppler. Thrombi can occur in any chamber but are most commonly ventricular [17].

Major risk factors include fetal vascular malperfusion—often related to cord or placental abnormalities—and fetal or maternal hypercoagulable states such as thrombophilia, diabetes, or lupus anticoagulant [18].

Outcomes vary with thrombus size, mobility, location, and comorbidities. In neonates, most thrombi resolve with anticoagulation, while infected thrombi require prolonged therapy or surgery. Mortality can reach 15%, particularly in preterm infants or those with large/mobile thrombi. Although embolization risk is low, fetal demise, adverse perinatal outcomes, and neurodevelopmental sequelae are well documented, especially when vascular malperfusion, infection, cardiac dysfunction, or severe underlying disease is present [19].

In our case, the lack of risk factors and the stable imaging over many weeks made a thrombus unlikely.

In the case we described, the right atrial mass was more consistent with HCT due to the following features: the echogenicity matched the adjacent atrial myocardium, the mass was located along the posterior/lateral wall between the SVC and IVC, the mass was stable with no growth over time, there was absence of internal flow or contrast enhancement, lack of hemodynamic effect, and stability in the post-natal period without modifications.

Given the wide range of possible explanations for intracardiac masses or variants, a structured and methodical imaging strategy is essential for accurate diagnosis and management.

First, serial imaging and growth assessment are fundamental: true tumors tend to enlarge over time during gestation, whereas benign anatomic variants typically remain static. In the present case, the critical finding of no change in size across follow-up scans strongly supported a non-neoplastic interpretation. Second, Doppler assessment of the lesion provides another valuable clue: internal vascular flow or contrast enhancement is characteristic of true tumors, whereas absence of flow strongly favors a nonvascular or structural variant. Here, the absence of internal flow reinforced the hypothesis of a benign anatomic structure. Third, a thorough hemodynamic evaluation is indispensable: one must look for any signs of obstruction (inflow or outflow), chamber dilatation, altered flows, hydrops fetalis, or functional compromise. The complete absence of any such findings in our case argued convincingly that the lesion was not exerting physiologically significant impact.

Detailed anatomical correlation and assessment of echogenic consistency further support the diagnosis: the lesion followed the expected SVC-to-IVC course along the right atrial wall, and its echogenicity matched surrounding atrial tissue—features well described as typical of this benign variant [8,9,20]. When standard imaging is inconclusive, advanced modalities such as fetal or neonatal MRI or postnatal TEE can aid tissue characterization or rule out alternative diagnoses. Several reports note that HCT can mimic a right atrial mass on 2D/3D echocardiography and that additional imaging (CT, MRI) can clarify the diagnosis [21,22].

Therefore, integrating the imaging findings into the broader clinical context is crucial: in the absence of extracardiac anomalies, hydrops, arrhythmia or other signs of systemic involvement, the suspicion for an isolated benign variant is substantially higher; genetic testing (where available) may further exclude syndromic associations. And lastly, postnatal correlation and longitudinal follow-up constitute the final and crucial step: confirmation after birth that the structure persists without growth, without new hemodynamic compromise or associated pathology effectively seals the benign nature of the variant. In our case, the postnatal echocardiographic series demonstrated persistence without change in size or function, thereby reinforcing the benign diagnosis.

By weaving together the absence of growth, the lack of vascular flow, the absence of hemodynamic disturbance, the anatomical course consistent with the CT, the supportive imaging context and the stable postnatal follow-up, the decision-making pathway clearly favored that this was a benign anatomic variant rather than a pathologic mass. This structured framework—serial growth assessment, Doppler evaluation, hemodynamic appraisal, anatomical correlation, advanced imaging when needed, contextual clinical synthesis and postnatal confirmation—offers a rigorous yet practical strategy for differentiating true tumors from benign variants in fetal and neonatal cardiac imaging.

By adding to the few documented cases in the literature, this report contributes valuable insight into the imaging characteristics, natural history, and clinical management of right atrial echogenic foci identified in fetuses. Better recognition of benign anatomic variants such as HCT may help prevent diagnostic confusion, unnecessary invasive testing, and undue parental anxiety [22,23]. This case highlights several key clinical lessons. When a right atrial echogenic focus is detected, particularly in late gestation, the CT should be included as part of the differential diagnosis.

Awareness of this normal structure, which runs from the SVC to the IVC along the lateral atrial wall, is essential to avoid mistaking it for a pathological mass [20,24]. When imaging features are reassuring—smooth, ridge-like morphology, echogenicity matching the atrial wall, and no internal vascular flow—and genetic testing is normal, conservative management with non-invasive serial surveillance is appropriate and avoids unnecessary invasive procedures.

In cases of diagnostic uncertainty, a multimodal imaging strategy is recommended. Two- and three-dimensional echocardiography with color Doppler offers detailed assessment of morphology, course, and vascularity, while fetal or neonatal MRI or postnatal TEE can further assist in tissue characterization and confirmation of benign anatomy [22,23]. Recent systematic reviews also show that multimodal imaging reduces misdiagnosis and supports the benign nature of HCT variants [1].

Parental counseling must be clear, including honest discussion of diagnostic certainty, reasons for conservative monitoring, and structured follow-up. Intervention should be limited to situations where concerning features—such as growth, obstruction, arrhythmia, or hydrops—develop. Sharing benign cases in the literature helps refine diagnostic algorithms and build evidence-based guidelines. Overall, the case described illustrates a balanced, cautious, and reassuring approach that avoids unnecessary intervention while maintaining appropriate vigilance.

Future research should focus on strengthening the evidence base for fetal CT variants through multicenter data collection, standardized echocardiographic protocols, and development of decision-support tools to distinguish benign variants from true pathology. Misdiagnosis of HCT as a cardiac tumor often leads to unnecessary imaging, surgical planning, or even oncologic evaluation, which can increase patient anxiety, procedural risks, and healthcare costs. Longitudinal studies are needed to determine whether prominent CT variants have long-term electrophysiologic effects. Incorporating advanced imaging—such as high-resolution echocardiography and fetal or neonatal MRI—may further improve diagnostic accuracy and reduce uncertainty [1,24,25].

## 4. Conclusions

Atrial structural variants should be included in the differential diagnosis of intracardiac masses, particularly when features favor stability and low risk. Serial imaging, avoidance of premature invasive measures, and careful counseling are key to appropriate management.

## Data Availability

The data presented in this study are available on request from the corresponding author.
